# Philadelphia Chromosome as a Clinically Favorable Prognostic Factor of B-cell Acute Lymphoblastic Leukemia/Lymphoblastic Lymphoma in Transplant-Ineligible Elderly Patients in the Era of Molecular-Targeted Therapy

**DOI:** 10.7759/cureus.73988

**Published:** 2024-11-19

**Authors:** Masuho Saburi, Takumi Nishikawa, Kuniaki Maehara, Keiichi Uraisami, Hiroyuki Takata, Yasuhiko Miyazaki, Kumiko Narahara, Hitohiro Sasaki, Miyuki Abe, Kazuhiro Kohno, Toshiyuki Nakayama, Eiichi Ohtsuka

**Affiliations:** 1 Department of Hematology, Oita Prefectural Hospital, Oita, JPN; 2 Department of Hematology, Oita Kouseiren Tsurumi Hospital, Oita, JPN

**Keywords:** b-cell all (acute lymphoblastic leukemia), blinatumomab, inotuzumab ozogamicin, philadelphia chromosome negative, philadelphia chromosome positive, tyrosine kinase inhibitor (tki)

## Abstract

Background and objective

There is scarce data on the treatment outcomes of B-cell acute lymphoblastic leukemia/lymphoblastic lymphoma (B-ALL/LBL) in elderly patients in the era of tyrosine kinase inhibitors (TKIs), blinatumomab, and inotuzumab ozogamicin. In light of this, we aimed to address this gap in data by conducting this retrospective study.

Methods

Treatment outcomes were retrospectively evaluated by using data from transplant-ineligible patients aged 65 years or older with newly diagnosed B-ALL/LBL (n=29) at two hospitals in Oita, Japan between 2013 and 2023.

Results

The median age of the cohort was 72 (65-88) years, and 10 patients were male; 17 patients had Philadelphia chromosome (Ph)-positive ALL, and the others had Ph-negative ALL. Dasatinib combined with prednisolone was the most common induction therapy for Ph-positive ALL (88.2%). Complete response (CR) was achieved in 93.1%, and the CR rate did not differ significantly between Ph-positive ALL (100%) and Ph-negative ALL (83.3%) (p=0.16). The median observation period was 1.52 (range: 0.03-8.98) years. Overall survival (OS) and event-free survival (EFS) were significantly longer in Ph-positive ALL patients than in Ph-negative ALL patients on univariate analysis (OS: p=0.011, EFS: p=0.041). Multivariate analyses showed that the presence of Ph was signiﬁcantly and independently associated with longer OS [hazard ratio (HR): 0.29, 95% confidence interval (CI): 0.10-0.87, p=0.027] and EFS (HR: 0.34, 95% CI: 0.12-0.91, p=0.03). There was no difference in relapse-free survival (RFS); 13 patients (76.5%) with Ph-positive ALL were treated with ponatinib (salvage therapy, n=7; consolidation or maintenance therapy in CR, n=6). Six of seven patients (85.7%) with ponatinib salvage therapy achieved CR, and all six patients treated with ponatinib consolidation or maintenance therapy retained CR at the last follow-up. Six patients (Ph-positive ALL: n=4; Ph-negative ALL: n=2) were treated with blinatumomab, including salvage therapy for primary refractory or relapse (n=3), and consolidation therapy due to intolerance to conventional chemotherapies (n=3). Two of three patients with blinatumomab salvage therapy achieved CR, and all three patients with blinatumomab consolidation therapy maintained CR in follow-up. Two patients (Ph-positive ALL: n=1; Ph-negative ALL: n=1) were treated with inotuzumab ozogamicin for relapsed or refractory ALL. A patient with Ph-positive ALL for the third relapse achieved CR, which was sustained for three years. The other patient with Ph-negative ALL for primary refractory achieved CR but relapsed after the fourth course of inotuzumab ozogamicin.

Conclusions

Elderly patients with Ph-positive ALL showed significantly longer OS and EFS than those with Ph-negative ALL in the era of molecular-targeted therapy.

## Introduction

Acute lymphoblastic leukemia (ALL)/lymphoblastic lymphoma (LBL) is the second most common acute leukemia in adults, and the hallmark of ALL is chromosomal abnormalities and genetic alterations involved in the differentiation and proliferation of lymphoid precursor cells. In adults, 75% of cases develop from precursors of the B-cell lineage, with the rest consisting of T-cell precursors [1]. B-cell lineage ALL (B-ALL) consists of subtypes with recurrent genetic alterations, represented by Philadelphia chromosome (Ph)-positive ALL [2]. Although Ph-positive ALL was initially seen as a factor associated with a poor prognosis, this view has changed with the improvement brought about by tyrosine kinase inhibitors (TKIs) [3] and allogeneic stem cell transplantation (allo-SCT) [4]. Also, it is now possible to cure Ph-negative ALL with negative measurable residual disease (MRD) without allo-SCT by intensive chemotherapy comprising vincristine, corticosteroids, anthracycline, cytarabine, methotrexate, and L-asparaginase [5,6].

Despite advances in treatment strategies for ALL, elderly patients often cannot tolerate intensive chemotherapies and have a worse prognosis. In recent years, several new molecular targeted therapies have been approved for relapsed or refractory ALL. Blinatumomab is a bispecific T-cell engager, which binds simultaneously to CD3-positive cytotoxic T-cells and CD19-positive B-cells, allowing the patient’s endogenous T-cells to recognize and eliminate CD19-positive B-ALL blasts [7,8]. Inotuzumab ozogamicin is an anti-CD22 antibody conjugated with calicheamicin. After the conjugate binds to CD22 of B-ALL, the CD22-conjugate complex is rapidly internalized, and calicheamicin is released. Calicheamicin binds to the minor groove of DNA and thus induces double-strand cleavage and subsequent apoptosis [9]. The third-generation TKI ponatinib has demonstrated efficacy not only in patients with refractory Ph-positive ALL with T315I mutant clones [10,11] but also in those with newly diagnosed Ph-positive ALL combined with reduced-intensity chemotherapy [12]. Recent treatment outcomes related to several new molecular-targeted drugs for elderly patients with B-ALL are unclear. Hence, this retrospective study aims to evaluate recent treatment outcomes of B-ALL/LBL in elderly patients.

## Materials and methods

This retrospective study included consecutive patients with newly diagnosed B-ALL/LBL aged 65 years or older and transplant-ineligible between 2013 and 2023 at two hospitals in Oita, Japan (Oita Prefectural Hospital and Oita Kouseiren Tsurumi Hospital). Patients who received no chemotherapy were excluded from this study. A total of 29 patients were enrolled. Ineligibility for transplantation was determined based on age (over 70 years), comorbidities, and individual patient preferences. Diagnosis of B-ALL was based on results of flow cytometry for CD45-blast gating with two-color analysis including CD2, CD3, cytoplasmic CD3, CD4, CD5, CD7, CD8, CD13, CD33, CD19, CD20, cytoplasmic CD22, CD56, cytoplasmic CD79a, cytoplasmic IgM, myeloperoxidase, and terminal deoxynucleotidyl transferase. LBL was diagnosed according to pathological B-cell phenotype lymphoid precursor mass with a percentage of bone marrow involvement of less than 25%.

Complete remission (CR) was defined as less than 5% blast cells in bone marrow for ALL and less than 5% blast cells in bone marrow without any extramedullary lesion for LBL. Relapse was defined as loss of CR. MRD was evaluated by reverse transcription-polymerase chain reaction (RT-PCR) for major or minor BCR-ABL using bone marrow specimens, which was performed at an outsourced laboratory, SRL. Patient data, including demographic characteristics, outcomes, and results of laboratory tests, were collected retrospectively from patient medical records.

Continuous variables were compared using the Student’s t-test or the Mann-Whitney U test as appropriate. Categorical variables were analyzed using Fisher’s exact test. Survival probabilities were estimated using the Kaplan-Meier method, and differences in survival distributions were compared using the log-rank test. Overall survival (OS) was calculated from the ALL/LBL diagnosis date to the date of death or last follow-up. Event-free survival (EFS) was calculated from the date of ALL/LBL diagnosis to the date of induction treatment failure, relapse from CR, or death from any cause. Relapse-free survival (RFS) in patients achieving CR after initial induction chemotherapy was measured from the date of achievement of CR to the date of relapse or death from any cause. Factors affecting OS, EFS, and RFS were analyzed using the log-rank test in univariate analyses and a Cox proportional hazards model in multivariate analyses.

The following variables were considered in the analysis of prognostic factors: age; sex; performance status (PS); white blood cell (WBC) and serum lactate dehydrogenase (LDH) at ALL/LBL diagnosis; and presence of Ph. Variables with p values <0.2 on univariate analyses were entered into multivariate analyses. For all analyses, p-values were two-sided, and p<0.05 was considered significant. All statistical analyses were performed with EZR [13]. CTCAE Ver5.0 eas used to evaluate adverse events. This consent procedure was reviewed and approved by the Ethics Review Board of Oita Prefectural Hospital. Opt-out informed consent protocol was used for the use of participant data for research purposes.

## Results

The characteristics of the cohort are presented in Table 1. The median age of the cohort was 72 (65-88) years, and 10 patients were male. Seventeen patients had Ph-positive ALL (58.6%), and the others had Ph-negative ALL/LBL including B-ALL, not otherwise specified (NOS) (n=10), B-ALL with KMT2A-AFF1 (n=1), and B-LBL (n=1). Twenty-six patients had pre-existing medical conditions or comorbidities. Table 2 shows clinical characteristics according to the presence of Ph.

**Table 1:** Patient characteristics (n=29)

Characteristics	Values
Age, years, median (range)	72 (65–88)
Sex, n, male/female	10/19
Performance status, n, median (range)	1 (0–4)
White blood cell, /μL, median (range)	10,440 (980–790,500)
Lactate dehydrogenase, U/L, median (range)	821 (147–4,321)
Subtype, n	
Acute lymphoblastic leukemia	28
Lymphoblastic lymphoma	1
Karyotype, n	
t(9;22)(q34;q11.2)	17
Normal karyotype	3
t(4;11)(q21;q23)	1
Complex karyotype	4
Other nonspecific abnormalities	3
No available data	1
BCR-ABL1 breaking point, n	
p190/p210	13/4
Comorbidities, n	
Hypertension	16
Dyslipidemia	8
Orthopedic disorder	8
Diabetes mellitus	6
Malignant tumors other than leukemia	5
Cerebrovascular disorder	4
Angina pectoris	2
Arrhythmia	2
Cognitive dysfunction	2
Chronic kidney disease	1
Thoracic aneurysm	1
Thyroid disease	1
Tuberculous lymphadenitis	1

**Table 2:** Comparison of clinical characteristics according to Philadelphia chromosome abnormality P-value <0.05 was considered statistically significant ALL: acute lymphoblastic leukemia

Variables	Philadelphia chromosome-negative ALL (n=12)	Philadelphia chromosome-positive ALL (n=17)	P-value
Age, years, median (range)	73.5 (65–88)	72 (66–80)	0.44
Sex, male, n (%)	7 (58.3)	3 (17.6)	0.046
Performance status, median (range)	1 (0–3)	1 (0–4)	0.84
White blood cell, /µL, median (range)	7,780 (840–790,500)	10,440 (1,480–167,100)	0.56
Lactate dehydrogenase, U/L, median (range)	978 (198–4,321)	717 (147–3,688)	0.48

**Figure 1:**
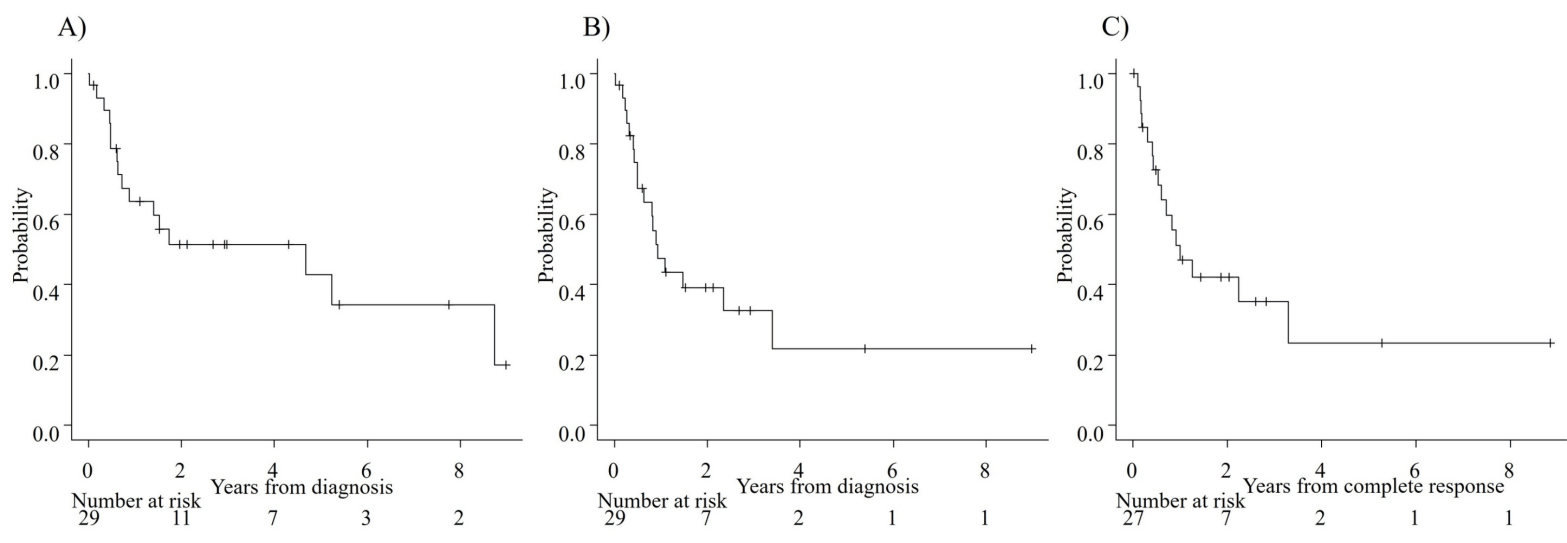
A) overall survival (OS), B) event-free survival (EFS), and C) relapse-free survival (RFS) The median (95% confidence interval) OS, EFS, and RFS are 4.67 (0.71-8.72) years, 0.93 (0.50-3.39) years, and 1.0 (0.54-3.29) years, respectively

**Figure 2:**
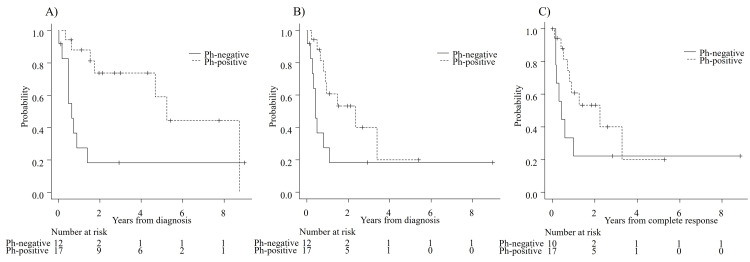
Comparison between patients with Philadelphia chromosome (Ph)-negative ALL and Ph-positive ALL A) overall survival (OS), B) event-free survival (EFS), and C) relapse-free survival (RFS). One-year OS and EFS are significantly higher for patients with Ph-positive ALL than for patients with Ph-negative ALL (OS: 87.8% vs. 27.5%, p=0.011; EFS: 60.8% vs. 27.5%, p=0.041). One-year RFS is not different between patients with Ph-positive ALL and Ph-negative ALL (60.8% vs. 33.3%, p=0.13). P-value <0.05 was considered statistically significant ALL: acute lymphoblastic leukemia

Females were common among patients with Ph-positive ALL, but this difference was not significant. Induction therapy was chosen as per the discretion of each attending physician as follows: dasatinib (n=15), imatinib (n=1), and CHOP (cyclophosphamide, doxorubicin, vincristine, and prednisone) (n=1) for Ph-positive ALL, and hyper-CVAD (cyclophosphamide, vincristine, doxorubicin, and dexamethasone) (n=7), VP (vincristine, prednisone) (n=2), prednisone (n=1), and an L-asparaginase regimen (n=2) for Ph-negative ALL/LBL. CR was achieved in 93.1%, and the CR rate did not differ significantly between patients with Ph-positive ALL (100%) and Ph-negative ALL (83.3%) (p=0.16). MRD was monitored by RT-PCR by using bone marrow specimens at the end of induction therapy (EOI) in 16 of 17 patients with Ph-positive ALL, and it was negative in 11 patients (68.7%) at EOI. MRD was not measured in any patients with Ph-negative ALL. The median follow-up duration after diagnosis was 1.52 (range: 0.03-8.98) years. The median OS was 4.67 [95% confidence interval (CI): 0.71-8.72] years, the median EFS was 0.93 (95% CI: 0.50-3.39) years, and the median RFS was 1.0 (95% CI: 0.54-3.29) years (Figures 1A-1C). Outcomes by the presence of Ph are shown in Figures 2A-2C.

Results of univariate and multivariate analyses for survivals are shown in Table 3. OS and EFS were significantly longer for patients with Ph-positive ALL than for patients with Ph-negative ALL on univariate analysis (one-year OS: 87.8% vs. 27.5%, p=0.011; one-year EFS: 60.8% vs. 27.5%, p=0.041); multivariate analyses showed that the presence of Ph was a significant, independent factor associated with longer OS [hazard ratio (HR): 0.29, 95% CI: 0.10-0.87, p=0.027] and EFS (HR: 0.34, 95% CI: 0.12-0.91, p=0.03). There was no difference in RFS, and no significant factors were found in the multivariate analysis for RFS. Two patients died within two months of the start of induction therapy. Both of these patients had primary refractory Ph-negative ALL and died of leukemia. Central nervous system involvement was observed in three patients with Ph-positive ALL at the time of relapse.

**Table 3:** Univariate and multivariate analysis of survival Variables with p-values <0.2 on univariate analyses were entered into multivariate analyses, and p-value <0.05 was considered statistically significant

Variables			Overall survival			Event-free survival			Relapse-free survival		
		Number of patients	Univariate	Multivariate		Univariate	Multivariate		Univariate	Multivariate	
			P-value	Hazard ratio (95% confidence interval)	P-value	P-value	Hazard ratio (95% confidence interval)	P-value	P-value	Hazard ratio (95% confidence interval)	P-value
Age	<75 years	17	0.29	- -	-	0.75	- -	-	0.93	- -	-
	≥75 years	12									
Sex	male	10	0.096	Reference 0.55 (0.19–1.61)	0.25	0.85	- -	-	0.54	- -	-
	female	19									
Performance status	0–1	15	0.59	- -	-	0.5	- -	-	0.81	- -	-
	2–4	14									
Philadelphia chromosome	Negative	12	0.011	Reference 0.29 (0.10–0.87)	0.027	0.041	Reference 0.34 (0.12–0.91)	0.03	0.14	Reference 0.47 (0.17–1.30)	0.15
	Positive	17									
White blood cell count	<10,000/µL	14	0.7	- -	-	0.26	- -	-	0.082	Reference 0.40 (0.13–1.16)	0.092
	≥10,000/µL	15									
Lactate dehydrogenase	<820 U/L	14	0.077	Reference	0.065	0.14	Reference		0.29	-	-
	≥820 U/L	14		2.78 (0.93–8.29			2.23 (0.82–6.08)			-	

Thirteen patients (76.5%) with Ph-positive ALL were treated with ponatinib. Five patients had a T315I mutation, and one of five patients had compound mutations of V299L and F317I. In patients treated with ponatinib, seven patients received salvage therapy for relapse, and six received consolidation or maintenance therapy in CR, including fou who received sequential therapy with TKIs and blinatumomab. Six of seven patients (85.7%) with ponatinib salvage therapy achieved CR, and all six patients treated with ponatinib consolidation or maintenance therapy retained CR at the last follow-up. No patients discontinued ponatinib due to adverse events. Adverse events (≥grade 3) of ponatinib were neutropenia (n=7), anemia (n=2), thrombocytopenia (n=4), liver dysfunction (n=1), hypertension (n=3), and infection (n=2). Heart failure (n=1) and sinus bradycardia (n=1) were observed as cardiovascular events, and both of them improved with temporary withdrawal of ponatinib. Six patients (Ph-positive ALL: n=4; Ph-negative ALL: n=2) were treated with blinatumomab. Of patients treated with blinatumomab, three received salvage therapy for primary refractory or relapse, and the others received consolidation therapy due to intolerance to conventional chemotherapy.

The median number of courses of blinatumomab was five (range: one to nine). Two of three patients receiving blinatumomab salvage therapy achieved CR, and all three patients receiving blinatumomab consolidation therapy maintained CR in follow-up. There was no obvious hematological toxicity, but hypogammaglobulinemia was observed in four patients (66.6%). Cytokine release syndrome (CRS) grade 1 was observed in two patients, but there was no immune effector cell-associated neurotoxicity syndrome (ICANS). Two patients (Ph-positive ALL: n=1; Ph-negative ALL: n=1) were treated with inotuzumab ozogamicin. A patient with Ph-positive ALL was treated for the third relapse after ponatinib treatment and achieved CR, which was sustained for three years. The other patient with Ph-negative ALL who was primary refractory after hyper-CVAD achieved CR, but relapsed after the fourth course of inotuzumab ozogamicin, and subsequently died of ALL. Sinusoidal obstruction syndrome (SOS) was not observed in the two patients.

## Discussion

In this retrospective study, the presence of Ph in B-ALL/LBL in elderly patients ineligible for transplantation in the era of molecular-targeted therapy was found to be an independent factor associated with longer OS and EFS. Age [14,15], WBC [1], and chromosomal abnormalities including Ph chromosome [16] were considered factors associated with a poor prognosis for B-ALL in the conventional chemotherapy era. Recently, genetic abnormalities such as Ph-like ALL [17,18] and IKZF1 mutation [19,20] have been reported as factors associated with a poor prognosis. Although these genetic abnormalities are more frequent in elderly patients, the intolerance to intensive chemotherapy due to organ dysfunction and comorbidities is an unmet medical need in elderly patients with ALL [21,22]. Therefore, advanced age is not only a factor in chemotherapy tolerability, but also in the biology of the disease, and therapeutic strategies focused on novel agents that are well tolerated and effective are key to improving outcomes.

In a single-center, retrospective study of elderly patients from 2000 to 2016 at the Mayo Clinic, despite high CR/complete remission with incomplete count recovery (CRi) rates, elderly ALL patients had a poor prognosis, and patients with Ph-positive ALL had a better prognosis [23]. No patients treated with ponatinib, blinatumomab, or inotuzumab ozogamicin were included in the Mayo Clinic study; thus our retrospective study is the first of its kind about the prognosis of B-ALL in transplant-ineligible elderly patients in the era of molecular-targeted therapy. TKIs greatly improved the prognosis of patients with Ph-positive ALL. Induction therapy with dasatinib showed a high CR rate, but disease-free survival (DFS) was 51.1% at 20 months with the appearance of T315I mutations in relapsed patients [24]. The GIMEMA LAL2116 (D-ALBA) trial evaluated the efficacy of chemotherapy-free treatment with dasatinib and blinatumomab, and it showed high MRD-negative rates with long-term progression-free survival (PFS) [25,26].

In the present study, sequential therapy with dasatinib and blinatumomab was performed in three patients unfit for conventional chemotherapy, and it resulted in durable CR with undetectable MRD. In addition, OS was prolonged, but not RFS. These results suggest that treatment with ponatinib after relapse improves OS, showing the importance of ponatinib in treating elderly patients with Ph-positive ALL. A phase 2 trial of the combination of ponatinib and blinatumomab for untreated Ph-positive ALL is being conducted at MD Anderson Cancer Center, with early reports of 40 patients showing good EFS and OS [27]. In the future, a chemotherapy-free regimen is expected to play an important role in improving the prognosis of patients with Ph-positive ALL, especially elderly patients. On the other hand, conventional chemotherapy remains the standard therapy for Ph-negative ALL. In our study, two patients died within two months of the start of induction therapy. Both patients had primary refractory Ph-negative ALL and died of leukemia. This may point to differences in EFS between patients with Ph-positive ALL and Ph-negative ALL.

Treatment strategies with blinatumomab and inotuzumab ozogamicin may be needed to improve the prognosis of Ph-negative ALL. The Blast TRIAL involving Ph-negative patients who were MRD-positive after induction chemotherapy showed a high MRD-negative rate of 78% and durable RFS [28-30]. The E1910 trial showed that the addition of blinatumomab to consolidation chemotherapy in adult patients in MRD-negative remission from B-ALL significantly improved OS [31]. Recently, a clinical trial of blinatumomab in newly diagnosed, elderly patients with Ph-negative ALL demonstrated a high CR rate and MRD-negative response (SWOG 1318, single-arm, phase 2 trial) [32]. First-line therapy with mini hyper-CVAD, blinatumomab, and inotuzumab ozogamicin resulted in longer EFS and OS than a historical control of hyper-CVAD in elderly patients with newly diagnosed Ph-negative ALL [33].

In the present study, blinatumomab was used in two patients with Ph-negative ALL. One of them was an 82-year-old elderly patient who was intolerant to initial chemotherapy in the first CR, and durable remission was observed with blinatumomab [34]. The other patient treated with blinatumomab had ALL with primary induction failures and died of ALL soon after blinatumomab administration. Currently, trials of induction with inotuzumab ozogamicin followed by consolidation with blinatumomab for older adults with newly diagnosed Ph-negative B-ALL (Alliance A041703, phase 2 study, NCT03739814) and of blinatumomab with chemotherapy vs. standard chemotherapy with newly diagnosed B-ALL (Golden Gate, phase 3 study, NCT04994717) are ongoing.

Limitations

The present study has several serious limitations: the retrospective nature of the study, the relatively small sample size, and the heterogeneity of the evaluated patients. Also, MRD was not measured in patients with Ph-negative ALL. MRD measurement in Japan has only been available in actual clinical practice since 2019.

## Conclusions

Our findings suggest that Ph is a significant, independent factor associated with longer OS and EFS in transplant-ineligible, elderly patients with B-ALL/LBL in the era of molecular-targeted therapies. Blinatumomab and inotuzumab ozogamicin-based chemotherapy-free regimens are expected to improve the treatment outcomes in patients with Ph-negative ALL.
